# Metastasis-inducing proteins are widely expressed in human brain metastases and associated with intracranial progression and radiation response

**DOI:** 10.1038/bjc.2016.103

**Published:** 2016-04-21

**Authors:** Rasheed Zakaria, Angela Platt-Higgins, Nitika Rathi, Daniel Crooks, Andrew Brodbelt, Emmanuel Chavredakis, David Lawson, Michael D Jenkinson, Philip S Rudland

**Affiliations:** 1 Department of Neurosurgery, The Walton Centre NHS Foundation Trust, Liverpool L9 7LJ, UK; 2 Institute of Integrative Biology, University of Liverpool, Liverpool L69 7ZB, UK; 3 Department of Neuropathology, The Walton Centre NHS Foundation Trust, Liverpool L9 7LJ, UK; 4 Institute of Translational Medicine, University of Liverpool, Liverpool L69 3BX, UK

**Keywords:** brain metastases, S100A4, osteopontin, biomarkers

## Abstract

**Background:**

Understanding the factors that drive recurrence and radiosensitivity in brain metastases would improve prediction of outcomes, treatment planning and development of therapeutics. We investigated the expression of known metastasis-inducing proteins in human brain metastases.

**Methods:**

Immunohistochemistry on metastases removed at neurosurgery from 138 patients to determine the degree and pattern of expression of the proteins S100A4, S100P, AGR2, osteopontin (OPN) and the DNA repair marker FANCD2. Validation of significant findings in a separate prospective series with the investigation of intra-tumoral heterogeneity using image-guided sampling. Assessment of S100A4 expression in brain metastatic and non-metastatic primary breast carcinomas.

**Results:**

There was widespread staining for OPN, S100A4, S100P and AGR2 in human brain metastases. Positive staining for S100A4 was independently associated with a shorter time to intracranial progression after resection in multivariate analysis (hazard ratio for negative over positive staining=0.17, 95% CI: 0.04–0.74, *P*=0.018). S100A4 was expressed at the leading edge of brain metastases in image guided sampling and overexpressed in brain metastatic *vs* non-brain metastatic primary breast carcinomas. Staining for OPN was associated with a significant increase in survival time after post-operative whole-brain radiotherapy in retrospective (OPN negative 3.43 months, 95% CI: 1.36–5.51 *vs* OPN positive, 11.20 months 95% CI: 7.68–14.72, Log rank test, *P*<0.001) and validation populations.

**Conclusions:**

Proteins known to be involved in cellular adhesion and migration *in vitro*, and metastasis *in vivo* are significantly expressed in human brain metastases and may be useful biomarkers of intracranial progression and radiosensitivity.

Brain metastases (BMs) are common brain tumours in adults with a steeply rising incidence due to the increased use of brain imaging in asymptomatic patients and prolonged survival from solid organ cancers (Owonikoko *et al*, 2014). There are no known biological markers that are routinely used to predict patient outcomes in BMs. Clinical factors are combined to generate predictions of overall survival (OS), but cannot predict intracranial progression, and the various models are not individualised to each patient, even if different primary cancer types are assessed separately (Sperduto *et al*, 2010).

We have previously identified two groups of proteins in the rat mammary model system that can induce metastasis and are associated with clinical outcomes in patients with breast (de Silva Rudland *et al*, 2011) and other solid organ cancers. S100A4 and S100P are small calcium-dependent regulatory molecules that are suggested to work by inducing cellular migration and invasion directly (Gross *et al*, 2014). S100A4 is active in the brain microenvironment (Dmytriyeva *et al*, 2012). Elevated levels of S100A4 are associated with a metastatic phenotype. It cooperates with growth-inducing activated oncogenes to yield growing metastases. Carcinomas in S100A4 knockout mice do not metastasise to the brain (Bresnick *et al*, 2015). The second group – osteopontin (OPN) and anterior gradient 2 (AGR2) – work primarily by inducing cellular adhesion to the extracellular matrix (ECM) that then allows migration to take place (Moye *et al*, 2004; Liu *et al*, 2005). Osteopontin binds the cell surface integrins *α*_V_*β*_3_/*α*_V_*β*_5_ with the latter widely expressed in human BMs and their microenvironment (Schittenhelm *et al*, 2013; Berghoff *et al*, 2014). The integrin *α*_V_*β*_3_/*α*_V_*β*_5_ inhibitor cilengitide induces cellular detachment and apoptosis, and reduces proliferation in a panel of brain metastatic breast cancer cell lines (Lautenschlaeger *et al*, 2013). AGR2 has been shown to be necessary and sufficient for migration *in vitro* in a glioblastoma cell line (Hong *et al*, 2013). Finally, the underlying change that is believed to result in selection for overexpression of these metastasis-inducing proteins (MIPs) is a failure of double-stranded DNA repair in a progenitor cell and in the breast this process is identified by immunohistochemical loss of the Fanconi anaemia protein, complementation group D2 (FANCD2) (Rudland *et al*, 2010). Notably, other closely related proteins in this family (Fanconi anaemia protein, complementation group A and G) have recently been shown to be overexpressed in BMs compared with the primary breast carcinoma in paired human samples (Woditschka *et al*, 2014).

We therefore studied these MIPs in human BMs to investigate whether they are overexpressed and whether their expression may be useful markers of clinical outcomes such as survival and progression.

## Materials and methods

### Patients and specimens

Patients with a diagnosis of brain metastasis (BM) were identified from histopathology records between 2005 and 2012 at a single institution and formalin-fixed, paraffin-embedded specimens were obtained in 138 cases. Full clinical information was gathered and is summarised in Table 1. For validation and investigation of intra-tumoral heterogeneity, 24 consecutive patients were included who underwent neurosurgical resection of a solitary supratentorial metastasis in non-eloquent brain by image-guided craniotomy as part of their standard care from 2014 to 2015. Clinical details are listed in Supplementary Data (Supplementary Table S1) and surgical, magnetic resonance imaging (MRI) techniques have been described previously (Zakaria and Jenkinson, 2014). Ethical approval was granted for this study within the Walton Research Tissue Bank for which all patients undergoing surgery are asked to give written informed consent (NRES 11/WNo03/2). Further ethical approval for use of archival and primary breast carcinoma specimens was granted by the UK Health Research Authority (NRES 12/NW/0778).

### Immunohistochemistry

Histological sections were cut at 4 μm on 3-aminopropyltriethoxy (APES)-coated slides, dewaxed in xylene and rehydrated through graded ethanol to water. First, endogenous peroxidase activity in the tissue sections was blocked by immersing the slides in 100% methanol containing 0.05% (v/v) H_2_0_2_ for 20 min at room temperature. Sections were then incubated in a moisture chamber with antibodies diluted in phosphate-buffered saline containing 1% (w/v) bovine serum albumen pH 7.4 as described for each stain further in Supplementary Data.

### Assessment of staining

Slides were analysed independently by two observers using light microscopy (RZ and NR) and corroborated by a senior neuropathologist (DC). The percentage of nuclear- and/or cytoplasm-stained tumour cells was recorded from well-separated sections of each specimen, 10 fields per section at × 200 magnification, at a minimum of 200 cells per field in a rigorous manner as described previously (Wang *et al*, 2006). There was agreement on positive staining (1% or above of cells positively stained to any degree (de Silva Rudland *et al*, 2011)) in 94% of slides scored, with a kappa statistic of 0.884. Slides were photographed using a Leica DFC310FX camera attached to a DM2000 microscope with the LAS V3 software suite (Leica Microsystems, Wetzlar, Germany; 2014) with no additional filtering or post processing of images.

### Statistical methods

Time from surgery to death was recorded as overall survival (OS) and non-cancer deaths or those lost to follow-up censored at last recorded follow-up. Progression-free survival (PFS) was recorded as time from surgery to documented intracranial progression as assessed by neuroradiologists using standard RANO (response assessment in neuro-oncology) criteria (Quant and Wen, 2011). Patients who died before this point were censored at the last date of follow-up where there was no evidence of progression. Proportions were assessed using Fisher's two-sided exact test. Time-to-event comparisons were made using Kaplan–Meier survival analysis with Log rank tests and multivariate analyses conducted using Cox's method. Data processing was performed using SPSS version 22.0 (IBM, Chicago, IL, USA) and R version 3.10 (R Core Team, 2013).

## Results

### Exploratory immunohistochemical staining

**Figure 1:**
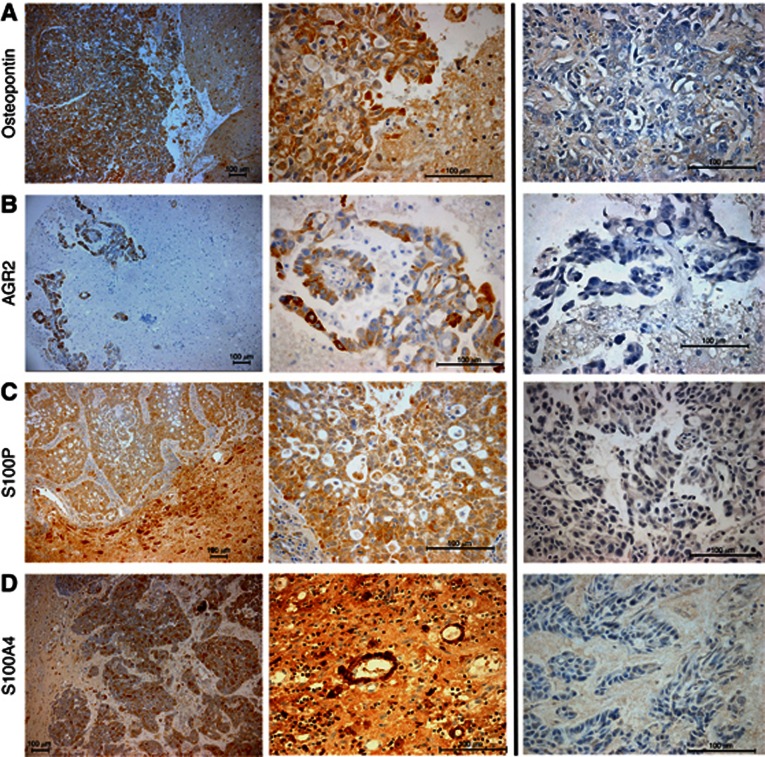
**Staining for the metastasis-inducing proteins in human brain metastases.**(A) Osteopontin in the tumour cytoplasm of a lung adenocarcinoma metastasis with some staining of the neuropial material in adjacent white matter. White matter and microglia, and astrocytes were easily distinguished morphologically from tumour cells and their staining was not counted when scoring slides. (B) AGR2 staining was seen mainly in the cytoplasm with no uptake in surrounding white matter as shown in this lung adenocarcinoma metastasis. (C) S100P staining in a lung adenocarcinoma with adjacent white matter shown – this protein, as in previous studies, was overexpressed in connective tissue and smooth muscle. (D) Nuclear and cytoplasmic staining for the protein S100A4 is shown in a brain metastasis from a breast carcinoma with avid staining of the endothelium also demonstrated. Taken at × 100 and × 400 magnification with scale bars shown (100 μm) and antigen-blocked immune serum controls given alongside.

Of 138 BMs assessed retrospectively, 16 were negatively stained for OPN (11.6%) and 122 (88.4%) were positively stained in varying proportions and intensities. This staining was mainly cytoplasmic with a stippled pattern, although some nuclear staining was also noted (Figure 1A). For AGR2 38 (27.5%), BMs were negatively stained, whereas 100 (72.5%) showed cytoplasmic staining. BMs from the posterior fossa that included cerebellar cortex showed incidental positive staining of what appeared to be the granule cells, but this did not affect the tumour staining analysis (Figure 1B). Assessment for S100P staining was positive (nuclear and cytoplasmic) in 102 BM (73.9%) cases, negative in 36 (26.1%). In areas of white matter adjacent to tumour, occasional astrocytes were seen to stain with anti-S100P antibody (Figure 1C); however, morphology and staining of serial sections with GFAP clarified that these were not tumour cells, thus avoiding any false positives. Glial staining for the purposes of this study was not considered further. For S100A4, 32 BMs (23.2%) were negative and 106 (76.8%) stained to some degree (Figure 1D). Staining was both nuclear and cytoplasmic; however, smooth muscle and endothelium were also seen to stain avidly with this antibody as noted previously. There was no staining of astrocytes nor peritumoral staining for S100A4 or OPN (Figure 1A and D). The heterogeneity of tissue staining was better appreciated in lower power micrographs (Supplementary Data; Supplementary Figure S1). There was no staining with antigen-blocked immune serum (Figure 1) nor with non-immune serum (Supplementary Data; Supplementary Figure S2) as negative controls. Melanoma cases required a different coloured chromogen (Supplementary Data; Supplementary Figure S3). The majority of BMs (113 or 81.9%) showed no immunoreactivity for FANCD2: only 25 (18.1%) showed weak cytoplasmic staining and there was no nuclear staining in any cases.

### Association between MIPs and primary cancer type: clinical features

**Figure 2:**
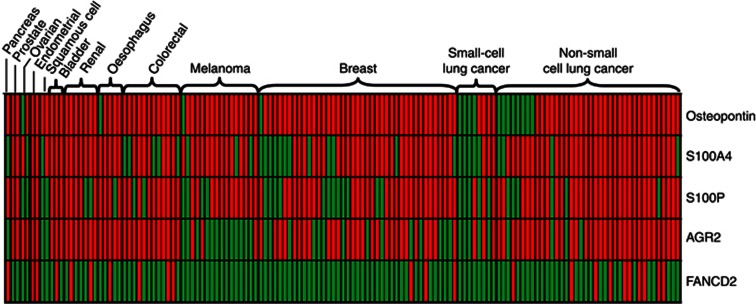
**Binary heat map showing the immunohistochemical staining of 138 brain metastases removed at neurosurgery for the metastases-inducing proteins osteopontin (OPN), S100A4, S100P, anterior gradient 2 (AGR2) and FANCD2.**Brain metastases are grouped by the primary cancer of origin with red squares showing positive staining of any degree (⩾1% carcinoma cells stained) and green squares indicating negative staining.

Figure 2 and Supplementary Data (Supplementary Table S2) show positive BM staining for each metastasis-inducing protein (MIP) by primary cancer type. There was no significant variation in BM staining for the S100 proteins by primary cancer (Fisher's exact test for S100P, *P*=0.279, and S100A4, *P*=0.135). There were significantly more AGR2-positive colorectal and non-small cell lung cancer BMs than expected (*P*<0.001), but fewer OPN-positive lung cancer BMs of all types (*P*=0.033). Importantly, none of the clinical features that are traditionally used to determine prognosis in patients with BMs (Gaspar *et al*, 1997b; Sperduto *et al*, 2008) were associated with positive staining for any of the MIPs (summarised in Supplementary Data and Supplementary Table S3).

### Association of MIPs with patient outcomes

**Figure 3:**
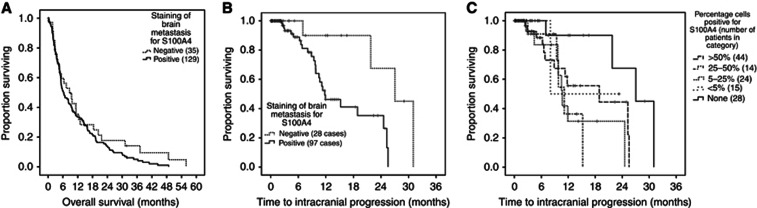
**(A) Survival of patients with and (B, C) disease progression of 138 brain metastases from different primary sites.**(**A**) Proportion of patients surviving is plotted against overall survival time as. Kaplan–Meier curves for positive (>1% carcinoma cells stained) and negative (<1% carcinoma cells stained) immunohistochemically stained brain metastases for S100A4. Survival time was not significantly associated with staining for S100A4 (Log rank test, *P*=0.222). (**B**) Proportion of patients surviving without intracranial progression is plotted against time as Kaplan–Meier curves for positive and negative immunohistochemically stained brain metastasis for S100A4. These patients had a grossly resected tumour. Median time to progression was significantly shorter in cases staining positive for S100A4 (11.77 months, 95% CI: 7.07–16.47) *vs* negatively stained cases (27.03 months, 95% CI: 16.49–37.57), Log rank test, *P*=0.007. This effect persisted in multivariate Cox analysis (HR 0.166, 95% CI: 0.04–0.74, *P*=0.018). (**C**) S100A4-positive cases in **B** above are subdivided into categories by the proportion of carcinoma cells in the specimen staining to various degrees for the S100A4 protein (pooled Log rank test (4 df)=9.806, *P*=0.044). Ticks indicate censored data in all panels.

Median OS was 7.67 months (95% CI: 4.45–10.89) and only age<60 years (HR=0.56, 95% CI: 0.33–0.94, *P*=0.028) was found to be independently associated with prolonged OS. There was no relation between positive MIP staining and OS (Figure 3A; Supplementary Data; Supplementary Table S4). Among patients receiving adjuvant whole-brain radiotherapy (WBRT), OS was 3.43 months (95% CI: 1.36–5.51) for OPN-negative cases, but 11.20 months (95% CI: 7.68–14.72) for positive cases, Log rank test, *P*<0.001. There was no confounding difference in age (Student's *t*-test, *P*=0.118), performance status (*P*=0.331) nor other clinical factors such as radioresistant tumour types (e.g., renal cancer BMs) between the groups to explain this effect. Different cutoffs for positive staining were used to check whether the percentage of tumour cells staining positive related to response to WBRT. There was a non-significant trend to prolonged median OS after WBRT with increasing percentage of positively OPN-stained tumour cells: 11.2 months if >5%, 13.9 months if >25% and 15.9 months if >50% positively stained.

Thirty solitary metastases that were completely resected showed intracranial progression at a median of 18.9 months from surgery (95% CI: 6.54–31.26). Table 2 lists the clinical factors associated significantly with prolonged PFS alongside MIP staining. As illustrated in Figure 3B, negative staining for S100A4 in the resected BM was the only factor independently associated with a longer PFS (HR for intracranial progression=0.17, 95% CI: 0.04–0.74, *P*=0.018). Tumour heterogeneity was assessed using different cutoffs for positive staining (see Supplementary Data and Supplementary Figure S1 for examples) and there was no difference in clinical factors or outcomes when assessing tumours with >5, >25 or >50% of S100A4-positive staining cells, illustrated for PFS in Figure 3C.

### Subtypes of BMs from common primaries

**Figure 4:**
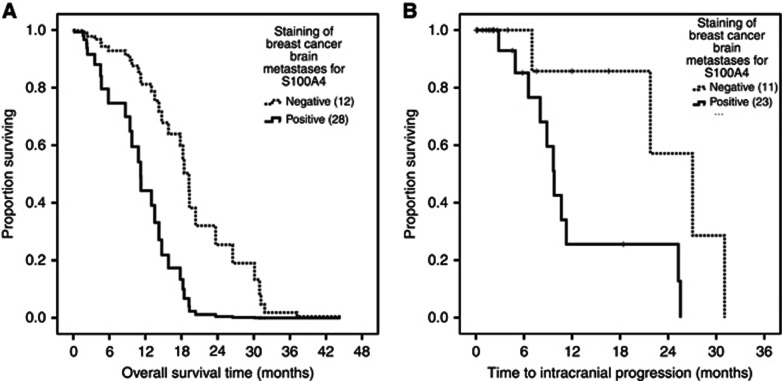
**(A) Survival of patients with and (B) disease progression of 40 brain metastases from primary breast cancer stained for S100A4.**(**A**) Proportion of patients surviving is plotted against overall survival time as Kaplan–Meier curves for positive (>1% carcinoma cells stained) and negative (<1% carcinoma cells stained) immunohistochemically stained brain metastases for S100A4. Positive staining in the brain metastasis was significantly associated with shorter overall survival in multivariate (Cox) analysis (HR of 0.26, 95% CI: 0.08–0.80, *P*=0.019) adjusted for age using the average covariate method (Makuch, 1982). (**B**) Proportion of patients surviving without intracranial progression is plotted against time to intracranial progression as Kaplan–Meier curves for positive and negative immunohistochemically stained brain metastases for S100A4. Fifteen out of 40 developed intracranial progression and of these, 11 out of 15 cases that were positively stained for S100A4 showed significantly earlier progression (median 9.77 months, 95% CI: 8.28–11.25) than the 4 negatively stained cases (median 27.03 months, 95% CI: 18.46–35.60, Log rank test, *P*=0.023). Ticks indicate censored data in all panels.

Forty patients with breast cancer were assessed separately and staining by subtype of breast carcinoma is shown in Supplementary Table S5. The median OS was 14.23 months (95% CI 9.21–19.26) and negative staining for S100A4 was independently associated with longer OS (HR for death=0.26, 95% CI: 0.08–0.80, *P*=0.019; Figure 4A) along with age <60 years (HR=0.30, 95% CI: 0.11–0.81, *P*=0.017) and post-operative chemotherapy (HR=0.12, 95% CI: 0.02–0.61, *P*=0.010). As an additional check, when the disease-specific graded prognostic assessment (DS-GPA) factors (Sperduto *et al*, 2010) for breast BM (age, subtype of carcinoma and performance status) were combined in a model, the predictive value of staining for the protein persisted (HR for death in S100A4-negative cases=0.58, 95% CI: 0.35–0.96, *P*=0.033). Intracranial progression occurred in 15 out of 40 breast carcinoma patients and the 11 out of 15 S100A4-positive cases showed significantly earlier intracranial progression (median 9.77 months, 95% CI: 8.28–11.25) than the 4 out of 15 negatively stained cases (median 27.03 months, 95% CI: 18.46–35.60, Log rank test, *P*=0.023; Figure 4B).

Non-small cell lung cancer patients had a median OS of 6.43 months (95% CI: 3.45–9.43) and 27 out of 38 received WBRT, this being the only factor associated with increased OS (HR of death if WBRT omitted=3.07, 95% CI: 1.08–8.69, *P*=0.035) regardless of incorporating MIP staining or the DS-GPA factors. Only 5 out of 38 patients developed intracranial progression – reflecting the burden of systemic disease on survival in these cases – but notably all of those BMs stained positively for S100A4.

There were 16 malignant melanoma cases and their median OS was 5.53 months (95% CI: 0.10–16.90). Incorporating the DS-GPA factors (number of BMs and performance status) with MIP staining showed that positive staining for S100A4 in the BM (13 out of 16 cases) was the only factor independently associated with decreased OS (HR for death in negatively stained cases=0.09, 95% CI:0.01–0.97, *P*=0.047). Only 5 out of 16 patients developed intracranial progression, and notably all of the S100A4-positive BMs progressed.

### Validation and investigation of intra-tumoral heterogeneity

Unselected BM samples from 24 prospectively treated patients were analysed, taking 1% as the cutoff for positive staining; 88% were S100A4 positive and 83% were OPN positive. This prospective validation cohort showed no significant differences from the retrospective cases in patient age, gender, size of operated metastasis, control of systemic disease, extracranial metastases or use of adjuvant chemo- and radiotherapy (Supplementary Data; Supplemetary Table S1). Nineteen out of 24 patients received adjuvant WBRT and, as in the retrospective series, this conferred a survival advantage in OPN-positive cases (6.3 months if irradiated *vs* 2.7 months if not, Log rank test, *P*=0.001) but not in OPN-negative cases (*P*=0.08). In total, 9 out of 24 cases showed intracranial progression and all of these were S100A4 positive (Supplementary Data; Supplementary Figure S4). In the course of resection, additional samples were obtained using image guidance at the leading edge of the BMs and all the MIPs showed a non-significant trend to a higher percentage of cells positive at the leading edge (Wilcoxon matched pairs analysis, *P*>0.05 for each MIP). S100A4 showed the greatest difference between percentage of positively staining cells at the edge and in the interior (ratio of 4.3 *vs* 1.7 for OPN, 2.5 for AGR2, 3.2 for S100P), although this ratio was not associated with any clinical outcome nor was it related to primary tumour type.

### Relationship of S100A4 staining to development of BMs

**Figure 5:**
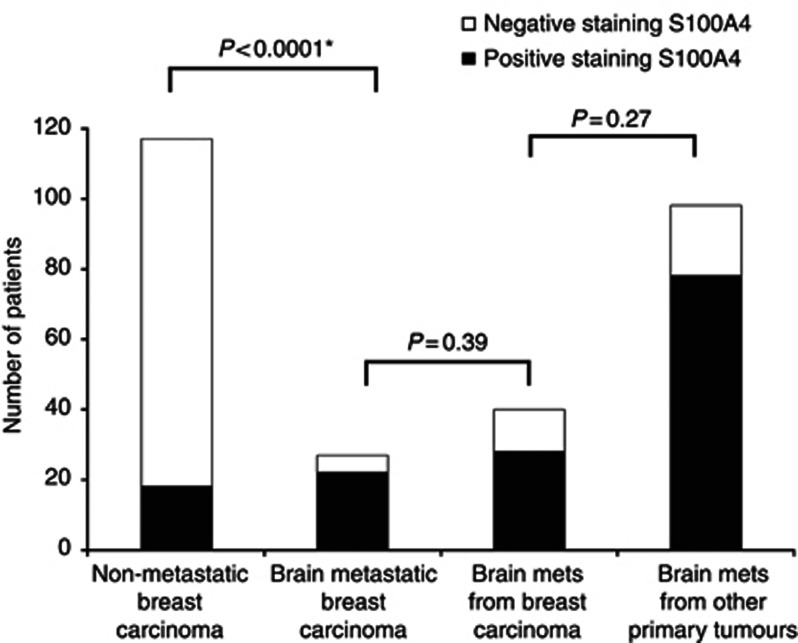
**Comparison of staining for S100A4 in non-metastatic and brain metastatic breast cancers.**The proportion of primary tumours staining positively for S100A4 in a group of previously reported patients (de Silva Rudland *et al*, 2011) with non-metastatic breast carcinoma surviving over 20 years was found to be significantly different from that of a group of breast carcinoma cases known to be brain metastatic (Fisher's exact test, *P*<0.0001). There was no significant increase in S100A4 positivity in the breast BMs themselves compared with the primary breast tumours nor in the proportion of S100A4-positive staining in BMs from other primaries compared with those from primary breast cancer (Fisher's exact test, *P*=0.39, *P*=0.27 respectively).

Given the relation of S100A4 overexpression to progression, the association of S100A4 with risk of BMs in patients with known cancer was investigated. In a series of breast cancer patients with BMs, 22 out of 27 of primary tumours (81%) were S100A4 positive compared with 18 out of 117 (15%) in a group with known non-metastatic breast cancer (Rudland *et al*, 2000) as shown in Figure 5 (Fisher's exact test, *P*<0.0001). The median time until development of BMs after diagnosis of breast cancer was 25.5 months (95% CI: 20.1–30.9) and was no shorter in the S100A4-positive cases (Log rank test *P*=0.67).

## Discussion

We have shown for the first time that proteins, which are (i) mechanistically proven to be involved in ECM adhesion and cell migration *in vitro*, (ii) convey a metastatic phenotype – including to brain – when overexpressed in animal models and (iii) are predictive of clinical outcomes in a variety of solid organ cancer cohorts, are also highly expressed at the protein level in human BMs and associate with important clinical outcomes. Previous publications have shown that the degree of immunohistochemical staining of carcinoma cells for the proteins described, OPN (Rudland *et al*, 2002), S100A4 (Rudland *et al*, 2000), S100P (Wang *et al*, 2006), AGR2 (Barraclough *et al*, 2009) and FANCD2 (Rudland *et al*, 2010), reflect the level of each particular protein in the specimens.

### Association of S100A4 with patient outcomes and possible clinical applications

We found comparable outcomes to other large, multicentre series of BM patients with age and performance status again shown to be strong predictors of OS (Gaspar *et al*, 1997a; Sperduto *et al*, 2008). In addition, we find that S100A4 was expressed in all progressing melanoma and non-small cell lung cancer BMs as well as being independently associated with time to intracranial progression in breast cancer – where patients had the longest OS time – but not in lung cancer, where patients were less likely to die from their brain disease. This result holds true even when known clinical predictors for each cancer type are incorporated into multivariate models (Sperduto *et al*, 2008) and suggests that S100A4 has some role in spreading in the brain microenvironment; in support of this suggestion, the protein was seen to be expressed at the leading edge of BMs in image-guided samples. It is known that S100A4 can reduce the formation of focal adhesions between cellular filopodia and the ECM via myosin heavy chain IIA to cause cell migration, invasion and metastasis (Gross *et al*, 2014), and thus it may represent a novel biological marker or a potential drug target. There is already interest in this protein as a monocloncal antibody target in metastatic melanoma and pancreatic cancer, following evidence that this family of proteins is a marker of aggressive, advanced tumours (Hernandez *et al*, 2013; Weide *et al*, 2013).

### Relationship of different proteins to BM development

Although staining for three MIPs is somewhat elevated in these BMs, only that for S100A4 shows a significant association with clinical outcomes in the form of time to intracranial progression in all BMs (Figure 3), and OS in breast cancer (Figure 4) and melanoma BMs. As positive staining for S100A4 occurs more often in advanced rather than in early breast cancers in contrast to the other three MIPs (de Silva Rudland *et al*, 2011; Winstanley and Rudland, 2013), it may be that only S100A4 has a role in the subsequent progression of those patients with BMs, whereas the other three MIPs stimulate earlier and different steps in the metastatic pathways. In support of this, we show for the first time that S100A4 is overexpressed in brain metastatic over non-metastatic breast cancers. Conversely, recent analysis of protein expression in the MDA-MB-231BR breast cancer cell line metastatic to mouse brains showed that S100A4 was under expressed compared to the parent MDA-MB-231 line (Dun *et al*, 2015). However, this report did not distinguish intra-from extracellular expression and was conducted on a triple negative cell line, whereas we found mostly HER2 and luminal subtypes in our patient group of BM. Moreover, as most of the proteome changes in 231 BR cells were decreases in individual protein levels, it is not clear whether the reduction in these proteins is important in metastasis or the proteins are downregulated because they have been selected against during the multiple cycles of injection and recovery from the immunosuppressed mice (Dun *et al*, 2015). The latter argument is more consistent with our earlier findings in thymectomised syngeneic rats and genetically immunesuppressed mice (Rudland *et al*, 1989).

### OPN as a marker of radiosensitivity

Although WBRT remains a pragmatic and readily available adjuvant treatment for BMs, there is concern regarding the cognitive effects in survivors and alternative post-operative management strategies are proposed. Using a simple BM marker to stratify patients as good or poor radiation, responders would therefore be an extremely useful clinical tool. Regarding therapeutics, cilengitide, an *α*v*β*3/*α*v*β*5 integrin inhibitor known to have efficacy in the brain microenvironment, appears to enhance radiation response in preclinical breast cancer BM models (Lautenschlaeger *et al*, 2013). It is therefore plausible that overexpression of OPN, an *α*v*β*3/*α*v*β*5 integrin ligand, in the BM may predict prolonged OS from adjuvant WBRT and this result merits further investigation.

### Limitations

Although retrospective data – particularly for performance status – is undesirable, a range of common cancers are represented in sufficient numbers to allow the lung, breast and melanoma to be explored separately and there were no missing data fields. To validate either protein as a clinical biomarker, a larger prospective study would be required recording tumour and possible also serum immunohistochemistry (IHC) levels of S100A4 and OPN alongside clinical outcomes (Dancey *et al*, 2010). MRI of asymptomatic patients at regular, e.g., two monthly follow-up, would have captured more detail on intracranial progression, reducing censored data in this category and clarifying if this were at the site of surgery, distant or leptomeningeal (an under-recognised phenomenon).

## Conclusions

Proteins known to be involved in cellular adhesion and migration *in vitro* and metastasis *in vivo* are significantly expressed in human BMs and may be useful biomarkers of intracranial progression and radiosensitivity.

## Figures and Tables

**Table 1:** Clinical details of retrospective series of patients studied

**Age at surgery (median, range)**	**59.9 years (20.3–82.4)**	
	**Number**	**Percentage total (%)**
**Karnofsky performance status**		
<70%	101	73.2
>70%	37	26.8
**Location of operated metastasis**		
Posterior fossa	33	23.9
Supratentorial	105	76.1
**Number of brain metastases**		
Multiple	26	18.8
Solitary	112	81.2
**Size of operated metastasis (diameter)**		
<30 mm	56	40.6
>30 mm	82	59.4
**Primary cancer controlled**		
No	29	29.9
Yes	68	70.1
**Extra-cranial metastases**		
Absent	91	65.9
Present	47	34.1
**Synchronous presentation: primary and brain metastases**		
No	97	70.3
Yes	41	29.7
**Primary cancer histology**		
Bladder	3	2.2
Breast	40	29
Endometrial	2	1.4
Colorectal	12	8.7
Renal	7	5.1
Melanoma	16	11.6
Non-small cell lung	38	27.5
Oesophagus	5	3.6
Ovarian	2	1.4
Pancreas	1	0.7
Prostate	2	1.4
Small cell lung	8	5.8
Squamous cell	2	1.4
**Type of operation**		
Biopsy	1	0.7
Gross total resection	127	92
Subtotal resection	10	7.2
**Whole-brain radiotherapy after neurosurgery^a^**		
No	33	23.9
Yes	105	76.1
**Chemotherapy after neurosurgery**		
No	86	62.3
Yes	52	37.7

a 30 Gy/5# most common.

**Table 2:** Clinical and biological factors associated with prolonged PFS time from resection to first brain progression of a metastasis

**Factor (events out of total)**	**Median PFS**/**months (95% CI)**	**Log rank comparison and significance**	**HR (95% CI) & significance in Cox regression**
**Age**			
<60 years (26 out of 63)	11.3 (3.49–19.1)	4.813, *P*=0.028*	0.97 (0.94–1.01), *P*=0.059
>60 years (4 out of 62)	Not reached		
**Performance status**			
KPS>70% (30 out of 90)	18.9 (6.54–31.26)	3.245, *P*=0.072	
KPS<70% (0 out of 35)	Not reached		
**S100A4 staining**			
Positive (26 out of 97)	11.77 (7.07–16.47)	7.295, *P*=0.007*	0.17 (0.04–0.74), *P*=0.018*
Negative (4 out of 28)	27.03 (16.49–37.57)		
**S100P staining**			
Positive (24 out of 95)	15.2 (6.16–24.25)	0.623, *P*=0.43	
Negative (6 out of 30)	24.57 (0–49.5)		
**AGR2 staining**			
Positive (20 out of 95)	21.77 (10.85–32.69)	1.117, *P*=0.291	
Negative (10 out of 30)	11.10 (8.06–14.14)		
**OPN staining**			
Positive (28 out of 110)	19.9 (6.25–31.5)	0.035, *P*=0.851	
Negative (2 out of 15)	15.2 (NA)		
**FANCD2 cytoplasmic staining**			
Positive (4 out of 23)	21.77 (6.88–36.66)	0.113, *P*=0.737	
Negative (26 out of 102)	15.20 (4.96–25.44)		

Abbreviations: AGR2=anterior gradient 2; CI=confidence interval; HR=hazards ratio; FANCD2=Fanconi anaemia protein, complementation group D2; NA, not applicable; OPN=osteopontin; PFS=progression-free survival.

*Significant relations are highlighted.
