# Supplementary material for: Metastasis-inducing proteins are widely expressed in human brain metastases and associated with intracranial progression and radiation response
Source: Br J Cancer. 2016 Apr 21;114(10):1101–8. doi: 10.1038/bjc.2016.103 (PMC4865966; doi:10.1038/bjc.2016.103)
Supplement: Supplementary Information [file bjc2016103x1.doc]

**Supplementary data**

**Methods**

*Immunohistochemistry*

Polyclonal affinity-purified anti-AGR2 (13) (#ARP42290, Aviva Systems Biology, San Diego, CA) was used at a dilution of 1:750 in phosphate-buffered saline (PBS) containing 2% (w/v) bovine serum albumen (BSA) pH7.4 and monoclonal anti-osteopontin (#MP111B10, Developmental Studies Hybridoma Bank, Iowa City, IA, USA) was used at 1:300 in 0.5% (w/v) BSA. Both were incubated on the sections overnight at 4^o^C. Polyclonal anti-S100A4 (#A5114, Dako UK Ltd, Ely, Cambridgeshire) and monoclonal anti-S100P (#610307, BD Biosciences, Oxford Science Park, Oxford) were used at a dilution of 1:400 and 1:75 respectively and were incubated for 2hr at room temperature, following an initial pre-incubation with 2% (w/v) BSA in PBS for 30 min at room temperature on the sections. Polyclonal anti-FANCD2 (#H300; sc28194, Santa Cruz Biotechnology Inc., Dallas, Texas, USA) was used at a dilution of 1:300 and was incubated for 3hr at room temperature, again following a 30 minute pre-incubation with 2% (w/v) BSA on the sections. FANCD2 antibodies recognize both the non-monoubiquitylated and monoubiquitylated isoforms of FANCD2 ^1^. Polyclonal anti-GFAP (#Z0334, Dako) and monoclonal anti-CD34 (#M7165, Dako) were used at a dilution of 1:2000 and 1:25 respectively and incubated on the sections for 1hr at room temperature. Prior to incubation, a 5 minute Proteinase K (Dako) digestion step was carried out by following the manufacturer’s instructions. After all the above antibody incubations, sections were thoroughly washed in three changes of PBS.

Indirect immunohistochemical staining was carried out using an enhanced horseradish peroxidase (HRP) labelled polymer system, the *DAKO EnVision+System, peroxidase(DAB)* ^2^ with reagents prepared according to the manufacturer’s instructions. After a final wash in running tap water, all sections were counterstained in Mayers' haemalum before being dehydrated through graded ethanol and xylene and mounted using DPX mountant (VWR, Lutterworth, Leicestershire).

For melanoma metastases a red colored chromogen (AEC)(Dako) was used to avoid confusion with pigmented, brown/black melanin-containing cells. This is illustrated in **Supplementary Figure S3**. In this case, the sections were mounted, without dehydration, using a water soluble mountant (Glycergel, Dako).

Non immune sera consisting of mouse IgG (Abcam) for mouse monoclonal antibodies (OPN and S100P) and rabbit IgG (Abcam) for rabbit polyclonal antibodies (S100A4 & AGR2) were substituted for primary antibodies and were included in each staining run. Breast carcinoma sections that were known to express all the proteins were used as external positive controls. Antigen-blocked immune serum was prepared by prior incubation with the appropriate recombinant protein as in previous studies on S100A4^3^, S100P^4^ and FANCD2 ^5^. Additionally AGR2 and OPN antibodies were blocked by pre-incubating it with the appropriate blocking peptides (# AAP42290 for AGR2and #AAP36677 for OPN, Aviva) in the ratio 100:1, Ag:Ab, with final antibody concentrations of 1:750 and 1:300 respectively. Blocking peptide pre-incubations were performed over 72hrs at 4°C. The control sera were then used in the immunohistochemical procedure as described above.

*References*

**1.** Wilson JB, Yamamoto K, Marriott AS, et al. FANCG promotes formation of a newly identified protein complex containing BRCA2, FANCD2 and XRCC3. *Oncogene.* Jun 12 2008;27(26):3641-3652.

**2.** Heras A ea. Enhanced labelled-polymer system for immunohistochemistry. *XVth Eur Cong Pathol.* Copenhagen, Denmark1995.

**3.** de Silva Rudland S, Martin L, Roshanlall C, et al. Association of S100A4 and osteopontin with specific prognostic factors and survival of patients with minimally invasive breast cancer. *Clinical cancer research : an official journal of the American Association for Cancer Research.* Feb 15 2006;12(4):1192-1200.

**4.** Wang G, Platt-Higgins A, Carroll J, et al. Induction of metastasis by S100P in a rat mammary model and its association with poor survival of breast cancer patients. *Cancer research.* Jan 15 2006;66(2):1199-1207.

**5.** Rudland PS, Platt-Higgins AM, Davies LM, et al. Significance of the Fanconi anemia FANCD2 protein in sporadic and metastatic human breast cancer. *The American journal of pathology.* Jun 2010;176(6):2935-2947.

**Figure S1:** Photomicrographs at lower magnifications (x12.5, x25 and x50) to demonstrate intratumoral heterogeneity of staining. Length of scale bars given in each panel.


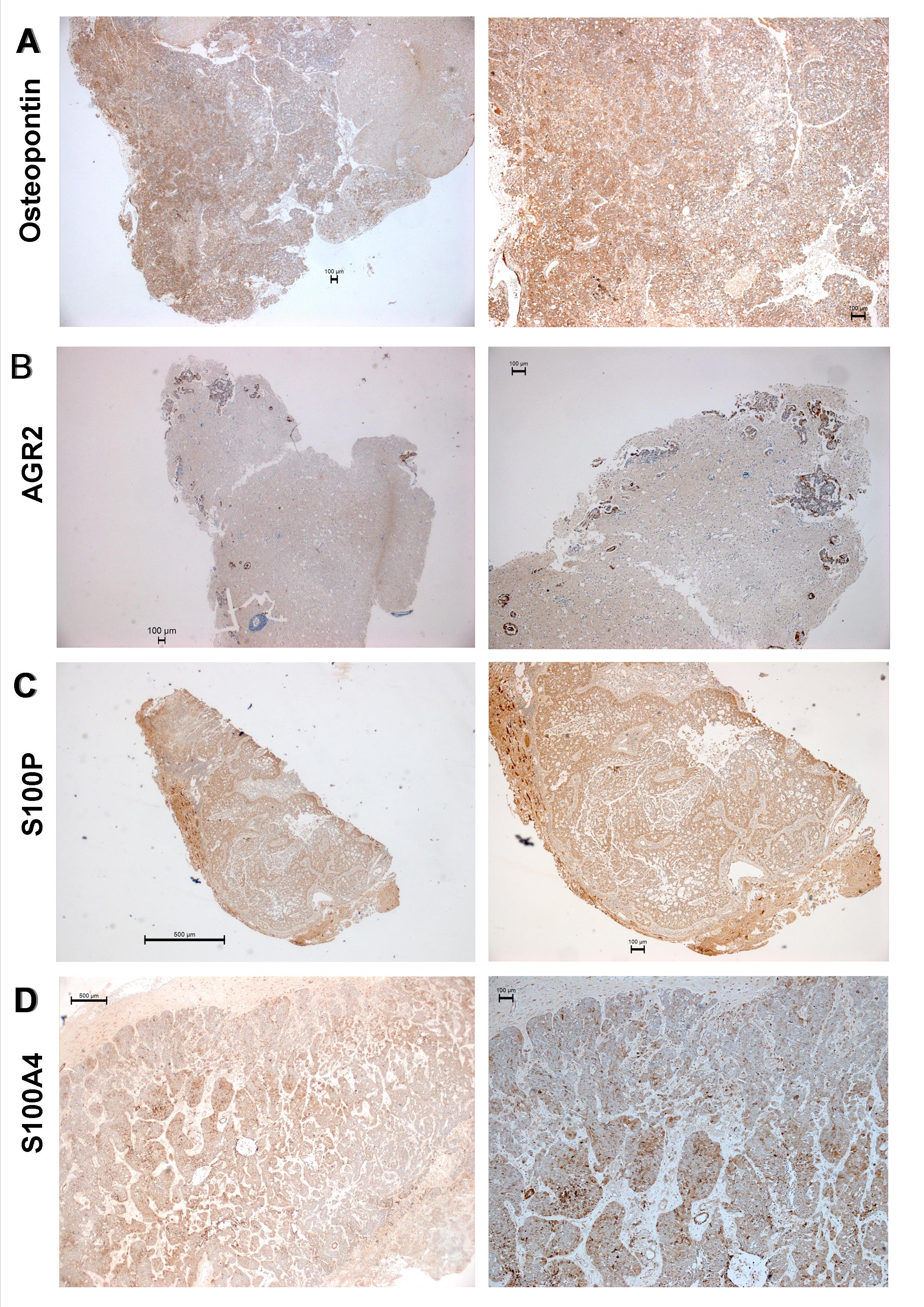


**Figure S2:** Negative controls: Non immune sera consisting of mouse IgG (Abcam) for mouse monoclonal antibodies (OPN & S100P) and rabbit IgG (Abcam) for rabbit polyclonal antibodies (S100A4 & AGR2) were substituted for primary antibodies and were included in each staining run. Photomicrographs at x100 and x400 magnification with 100µm scale bars shown.

**
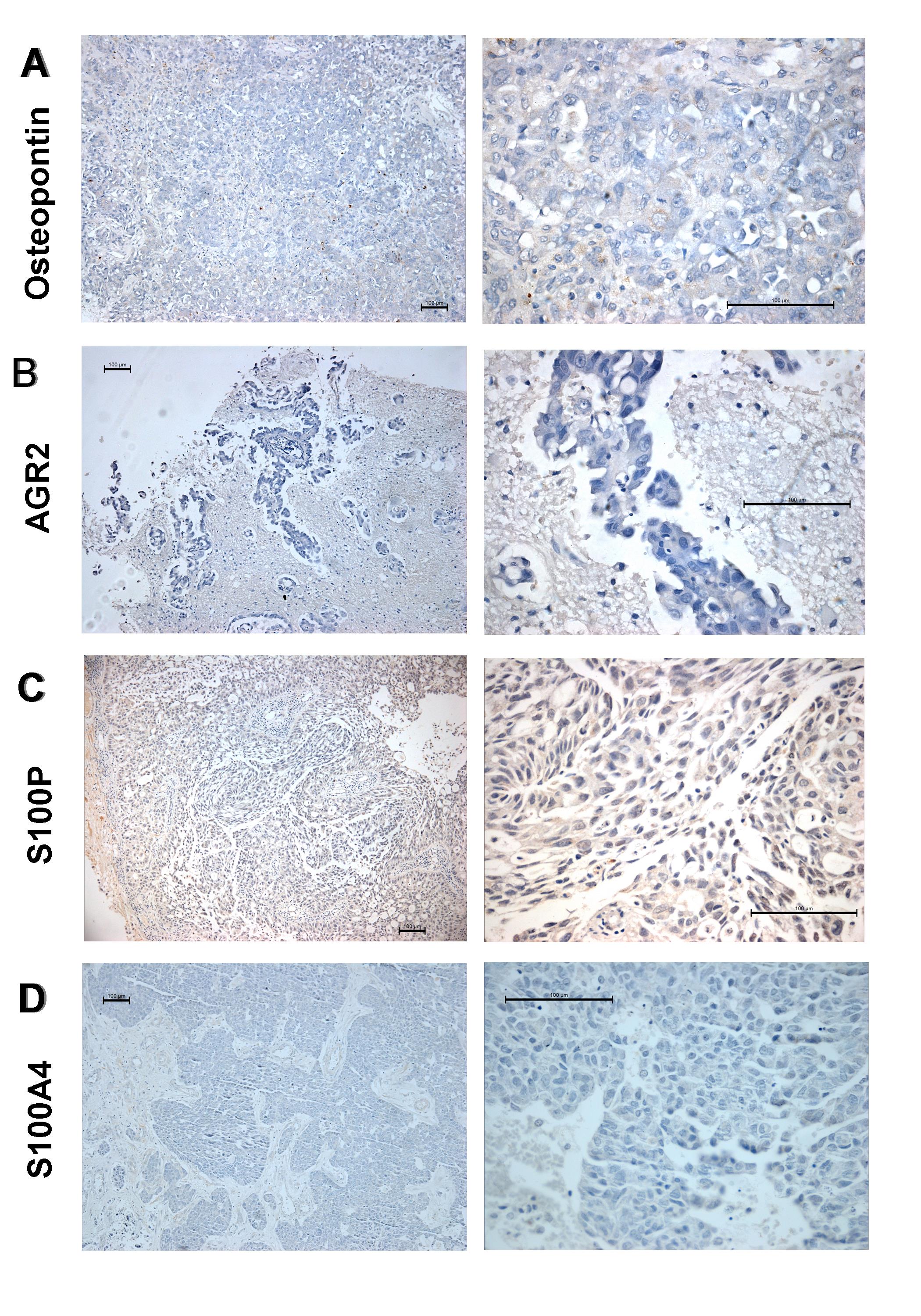
**

**Figure S3:** Immunohistochemical staining for the protein osteopontin in a 52 year old patient with metastatic melanoma who underwent craniotomy and resection. The H&E stained section (A) shows tumour cells with irregular, enlarged nuclei. Using standard antibody detection kits there seems to be evidence of protein expression in the cytoplasm predominantly (B), but the presence of brown and black melanocytes (C) makes it difficult to judge. Use of an alternative antibody detection kit with a red chromogen allows confirmation of staining (D) in tumour cells. Original magnification X 200, scale bars of 100 µm shown.

**
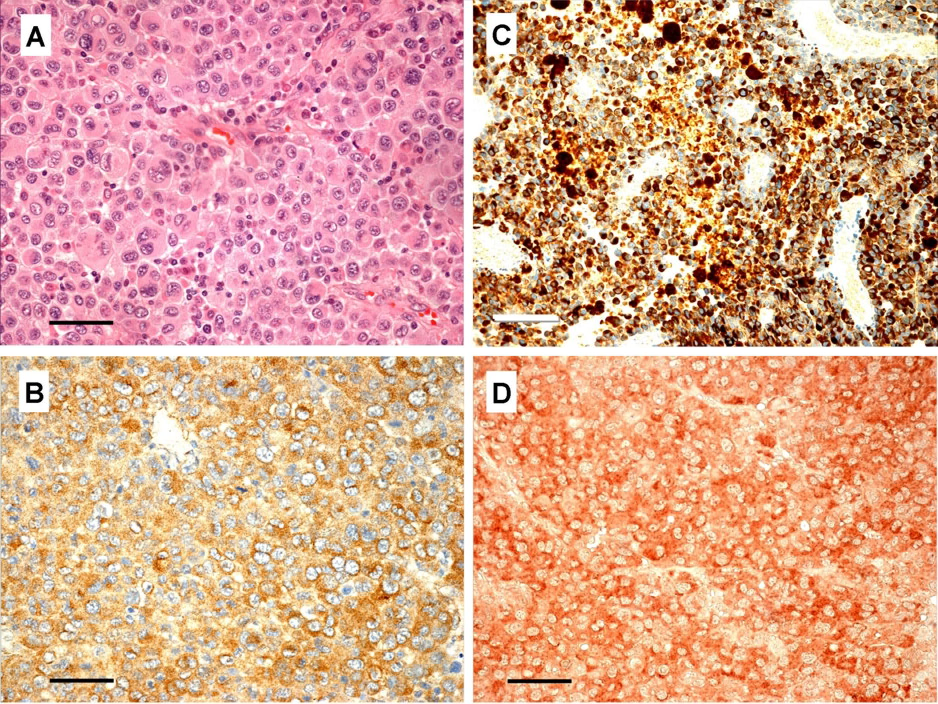
**

**Figure S4:** Kaplan-Meier plots showing time to intracranial progression for prospectively-analysed brain metastases of patients undergoing resection. All 21 cases with progression up to 13 months had stained positively for S100A4 although there was no significant difference in survival by the Log rank test (= 1.62, p = 0.203) compared to the 3 negatively stained cases.


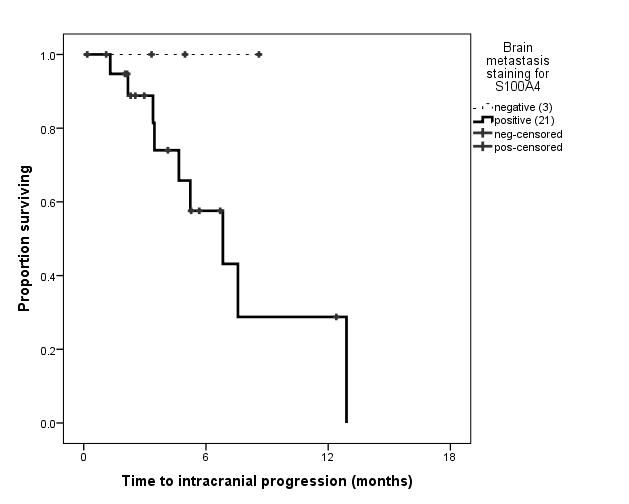


**Table S1:** Clinical data for prospective series of 24 patients with resected supratentorial metastases.

| **Age at surgery** | (median, range) | 59.3 years | (23.9 – 76.0) |
| --- | --- | --- | --- |
| **Gender** | Female | 13 | 54% |
|  | Male | 11 | 46% |
| **Primary carcinoma** | Non-small cell lung | 12 | 50% |
|  | Melanoma | 4 | 17% |
|  | Breast | 3 | 13% |
|  | Colorectal | 2 | 8% |
|  | Endometrial | 1 | 4% |
|  | Renal | 1 | 4% |
|  | Sarcoma | 1 | 4% |
| **Extracranial disease?** | Absent | 17 | 71% |
|  | Present | 7 | 29% |
| **Controlled primary?** | Synchronous presentation | 8 | 33% |
|  | Yes | 16 | 67% |
| **Adjuvant whole brain radiotherapy?** | No | 5 | 21% |
|  | Yes, 30 Gray in 10# | 19 | 79% |
| **Adjuvant chemotherapy?** | No | 13 | 54% |
|  | Yes | 11 | 46% |

**Table S2:** Table showing the proportion of brain metastases from different primary cancers staining positively (1% or above of cells positively stained to any degree) for each Metastasis Inducing Protein.

| Primary cancer and number of cases | | | **Osteopontin** | | **S100A4** | | **S100P** | | **AGR2** | |
| --- | --- | --- | --- | --- | --- | --- | --- | --- | --- | --- |
|  |  |  | negative | positive | negative | positive | negative | positive | negative | positive |
|  | **Breast** | 40 | 2% | 98% | 30% | 70% | 30% | 70% | 35% | 65% |
|  | **Non-small cell lung** | 38 | 21% | 79% | 11% | 89% | 16% | 84% | 11% | 89% |
|  | **Small cell lung** | 8 | 50% | 50% | 62% | 38% | 62% | 38% | 50% | 50% |
|  | **Melanoma** | 16 | 6% | 94% | 19% | 81% | 25% | 75% | 81% | 19% |
|  | **Colorectal** | 12 | 0% | 100% | 42% | 58% | 17% | 83% | 0% | 100% |
|  | **Renal** | 7 | 0% | 100% | 0% | 100% | 29% | 71% | 0% | 100% |
|  | **Oesophagus** | 5 | 20% | 80% | 0% | 100% | 20% | 80% | 0% | 100% |
|  | **Bladder** | 3 | 0% | 100% | 0% | 100% | 0% | 100% | 0% | 100% |
|  | **Squamous cell** | 2 | 0% | 100% | 50% | 50% | 100% | 0% | 100% | 0% |
|  | **Ovarian** | 2 | 50% | 50% | 50% | 50% | 100% | 0% | 0% | 100% |
|  | **Prostate** | 2 | 0% | 100% | 0% | 100% | 0% | 100% | 0% | 100% |
|  | **Endometrial** | 2 | 0% | 100% | 0% | 100% | 0% | 100% | 0% | 100% |
|  | **Pancreas** | 1 | 0% | 100% | 100% | 0% | 0% | 100% | 100% | 0% |

**Table S3:** Relation of clinical factors to positive staining for the Metastasis Inducing Proteins and cytoplasmic FANCD2 in 138 resected brain metastases cases.

| Factor & level | | **Osteopontin** | | **S100A4** | | **S100P** | | **AGR2** | | **FANCD2** | |
| --- | --- | --- | --- | --- | --- | --- | --- | --- | --- | --- | --- |
|  |  | negative | positive | negative | positive | negative | positive | negative | positive | negative | positive |
| **Metastasis location** | Post fossa | 9% | 91% | 18% | 82% | 36% | 64% | 18% | 82% | 76% | 24% |
|  | Supratentorial | 12% | 88% | 25% | 75% | 23% | 77% | 30% | 70% | 84% | 16% |
| **Number of metastases** | Multiple | 4% | 96% | 23% | 77% | 27% | 73% | 35% | 65% | 88% | 12% |
|  | Solitary | 13% | 87% | 23% | 77% | 26% | 74% | 26% | 74% | 80% | 20% |
| **Synchronous presentation** | No | 8% | 92% | 23% | 77% | 24% | 76% | 29% | 71% | 87% | 13% |
|  | Yes | 20% | 80% | 24% | 76% | 32% | 68% | 24% | 76% | 71% | 29%* |
| **Controlled primary** | No | 10% | 90% | 21% | 79% | 17% | 83% | 24% | 76% | 90% | 10% |
|  | Yes | 7% | 93% | 23% | 77% | 26% | 74% | 31% | 69% | 85% | 15% |
| **Extra-cranial metastases** | No | 14% | 86% | 22% | 78% | 25% | 75% | 23% | 77% | 78% | 22% |
|  | Yes | 6% | 94% | 26% | 74% | 28% | 72% | 36% | 64% | 89% | 11% |
| **Performance status** | >70% | 11% | 89% | 24% | 76% | 26% | 74% | 33% | 67% | 86% | 14% |
|  | <70% | 14% | 86% | 22% | 78% | 27% | 73% | 14% | 86%* | 70% | 30%* |

*indicates significantly different proportion positive:negative staining than expected, Fisher’s exact test, 2-sided, p<0.05

**Table S4:** Table showing the overall survival time for patients with a resected brain metastasis at different levels of common clinical factors and with positive or negative staining for the Metastasis Inducing Proteins and FANCD2. Log rank test statistics for comparison of survival (* = p<0.05) are given.

| Factor | Median overall survival/ months | 95% CI | Log rank & significance |
| --- | --- | --- | --- |
| Age  <60 years  >60 years | 13.0  3.77 | 9.16 – 16.84  2.48 – 5.06 | 17.549  p < .005* |
| Size of metastasis  Diameter<30mm  Diameter>30mm | 9.43  5.37 | 3.34 – 15.53  3.44 – 7.30 | 2.883  p =.09 |
| Location of metastasis  Supratentorial  Posterior fossa | 9.30  4.90 | 5.99 – 12.61  3.05 – 6.80 | 1.302  p=.254 |
| Number of metastases  Solitary  Multiple | 7.67  5.77 | 0 – 15.95  4.40 – 10.93 | 0.23  p=.879 |
| Synchronous presentation  Yes  No | 4.93  9.73 | 1.55 – 8.31  5.27 – 14.20 | 5.627  p=.018* |
| Neurosurgery procedure  Biopsy  Gross total resection vs.  Subtotal resection | not reached  8.27  2.93 | 4.79 – 11.74  0 – 6.08 | 9.131  p=.01* |
| Controlled primary disease  Yes  No | 13.97  4.53 | 10.24 – 17.7  3.84 – 5.22 | 6.753  p=.009* |
| Extra-cranial metastases  Present  Absent | 7.67  6.83 | 3.11 – 12.22  2.88 – 10.79 | .159  p=.69 |
| Performance status  KPS<70%  KPS>70% | 3.93  10.83 | 2.07 – 5.79  8.32 – 13.35 | 19.21  p=.000* |
| Adjuvant whole brain radiotherapy  Yes  No | 9.90  2.73 | 7.58 – 12.22  2.40 – 3.07 | 20.861  P<.005* |
| Adjuvant chemotherapy  Yes  No | 18.23  4.03 | 13.00 – 23.46  2.85 – 5.22 | 28.117  P<.005* |
| Primary (where enough cases for analysis)  Breast cancer vs.  Non-small cell lung cancer  or melanoma | 14.23  6.43  5.53 | 9.21 – 19.26  3.45 – 9.41  0 – 16.90 | 9.659  p=.022* |
| S100A4 staining  Positive  Negative  S100P staining  Positive  Negative  AGR2 staining  Positive  Negative  OPN staining  Positive  Negative  FANCD2 cytoplasmic staining  Positive  Negative | 6.83  9.50  7.40  8.37  6.83  9.43  9.30  3.37  6.83  8.23 | 5.59 – 13.41  3.51 – 10.16  3.07 – 11.73  3.63 – 13.10  3.93 – 9.74  3.62 – 15.25  6.29 – 12.31  2.06 – 4.67  2.37 – 11.29  4.94 – 11.52 | 1.21  p=.271  1.652  p=.199  .326  p=.568  6.861  p=0.09  .012  p=.912 |

**Table S5:** Staining of brain metastases for Metastasis Inducing Proteins and FANCD2 in patients with primary breast carcinomas by subtype.

| Breast Cancer | **Osteopontin** | | **S100A4** | | **S100P** | | **AGR2** | | **FANCD2** | |
| --- | --- | --- | --- | --- | --- | --- | --- | --- | --- | --- |
| Subtype (number) | negative | positive | negative | positive | negative | positive | negative | positive | negative | positive |
| **HER2 (18)** | 0% | 100% | 22% | 78% | 22% | 78% | 17% | 83% | 89% | 11% |
| **Triple negative (12)** | 0% | 100% | 33% | 67% | 50% | 50% | 67% | 33% | 92% | 8% |
| **Luminal (8)** | 0% | 100% | 38% | 63% | 25% | 75% | 25% | 75% | 100% | 0% |
| **Not specified (2)** | 50% | 50% | 50% | 50% | 0% | 100% | 50% | 50% | 100% | 0% |
